# Pharmaceutical Assessment of the Impact of the Method of Extraction on the Suitability of Pectin from Plantain (*Musa paradisiaca*) Peels as a Suspending Agent in Oral Liquid Formulations

**DOI:** 10.1155/2023/8898045

**Published:** 2023-09-29

**Authors:** Frederick William Akuffo Owusu, Prince George Jnr Acquah, Mariam E. L. Boakye-Gyasi, Raphael Johnson, Genevieve Naana Yeboah, Mary-Ann Archer, Mercy Birago Antwi, Sandra Obenewaa Asare

**Affiliations:** ^1^Department of Pharmaceutics, Faculty of Pharmacy and Pharmaceutical Sciences, Kwame Nkrumah University of Science and Technology, Kumasi, Ghana; ^2^Department of Pharmaceutics and Quality Control, Centre for Plant Medicine Research, Mampong, Ghana; ^3^Department of Pharmaceutics, School of Pharmacy and Pharmaceutical Sciences, University of Cape Coast, Cape Coast, Ghana

## Abstract

Natural polymers such as pectin have gained increased utilization in pharmaceutical and biotechnology sectors because they are affordable, easily accessible, nontoxic, and chemically modifiable, with the potential to be biodegradable and biocompatible. *Musa paradisiaca* (plantain) peels make up 30–40% of the overall weight of the fruit. The extraction of pectin from these residues can therefore be viewed as a possible waste of wealth. This study, therefore, focused on evaluating the suspending properties of pectin obtained from *Musa paradisiaca* (plantain) peels (through acid and alkaline extraction) and presented an alternative suspending agent in the pharmaceutical formulation of suspensions. The unripe peels of *Musa paradisiaca* were acquired and authenticated at the Department of Pharmacognosy, Faculty of Pharmacy and Pharmaceutical Sciences, Kwame Nkrumah University of Science and Technology (KNUST), Kumasi, Ghana. Pectin was extracted from the peels using both acid and alkaline extraction processes, respectively, characterized, and evaluated for its phytochemical properties. Different concentrations of the acid and alkaline pectin extracts were employed as a suspending agent in paracetamol suspensions, using acacia gum as a standard. The pectin yields obtained were 4.88% and 7.61% for the acid and alkaline extraction processes, respectively, while phytochemical screening revealed the presence of glycosides, tannins, saponins, and phenols in both extracts. The alkaline pectin extract recorded higher equivalent weight, degree of esterification, ash content, and crude content than the acid pectin extract, while FTIR identified similar functional groups in both acid and alkaline pectin extracts. The test suspensions reported significant differences (*P* < 0.05) in flow rates, ease of redispersion, sedimentation volumes, and rates compared with acacia gum. Moreover, when the acid and alkaline pectin extracts were compared, significant differences (*P* < 0.05) were observed in sedimentation rates and sedimentation volumes, suggesting that the extraction method may affect suspending properties. Ultimately, the alkaline pectin extract had better suspending properties than the acid pectin extract; however, they both can be used as an alternative to acacia gum as a suspending agent.

## 1. Introduction

Pharmaceutical suspensions are liquid dosage forms made up of insoluble solid particles dispersed in an aqueous or nonaqueous medium and tend to increase the bioavailability of active pharmaceutical ingredients compared to solid dosage forms such as tablets [1]. Oral suspensions are also convenient for children and the elderly with difficulty swallowing solid oral dosage forms such as capsules and tablets [1, 2]. Nevertheless, the thermodynamic instability of suspensions presents a significant limitation [3]. There is the sedimentation of insoluble active pharmaceutical ingredients upon storage; therefore, ease of dispersibility after shaking is a significant advantage of ideal suspensions, and this is achieved by adding suspending agents [4]. Polymers such as pectin have been routinely employed as suspending agents to increase viscosity and decrease the sedimentation rate of suspensions [4].

Pectin is an anionic structural heteropolysaccharide sizeable molecular weight polymer found abundantly in the primary cell wall of plants [5]. It is composed of *α*-(1–4)-D-galacturonic acid units, forming long homogalacturonic chains interspersed by rhamnogalacturonan components with alternating residues of rhamnose and galacturonic acid. Neutral sugar units are enriched in highly branched, “hairy” regions and are attached to the backbone. Portions of the galacturonic chain's carboxylic groups are present as methyl esters [6].

Pectin has been used successfully for many years as a thickening agent, a gelling agent, and a colloidal stabilizer in the food and beverage sector [7]. Commercially, it is made predominantly from apple pomace and citrus peel [5]. Pectin has also become more popular in pharmaceutical and biotechnology industries as binders, disintegrants, suspending agents, and biodegradable carriers for control-released drug delivery systems [8–10]. Natural polymers, such as pectin, are being used in pharmaceutical and biotechnology industries because they are affordable, easily accessible, nontoxic, and chemically modifiable [11].

Throughout the world, plantain is a significant staple item and can be used to prepare food at all ripening stages, even when overripe [12]. Peels from plantains make up 30–40% of the overall weight of the fruit. The extraction of pectin from these residues can therefore be viewed as a possible waste to wealth [13]. The extraction method and other factors affect the quality and suitability of pectin as a pharmaceutical excipient. Conventionally, alkaline or acid hydrolysis of protopectin followed by ethanol precipitation is preferred because of the cost and availability compared to other novel methods, such as microwave-assisted extraction [14]. It is generally reported that the pectin yield and neutral sugar side chains are increased by alkaline extraction. However, saponification and *β*-elimination reactions result in low methyl esterification and molecular weight of alkaline extracts compared with acid extracts. These parameters have been reported to affect the gelling properties of pectin and its subsequent pharmaceutical applications [15, 16].

The focus of this study was to evaluate the suspending properties of pectin obtained from *Musa paradisiaca* (plantain) peels using acid and alkaline extraction and present an alternative suspending agent in the pharmaceutical formulation of suspensions. This feat, if achieved, would further diversify the use of local plantain in Ghana and commodify the utilization of plantain peels by local pharmaceutical manufacturers in Ghana.

## 2. Materials and Methods

### 2.1. Materials

Paracetamol powder (99%) was purchased from Xi'an Henrikang Biotech Co., China, and acacia powder was purchased from Sigma-Aldrich, Darmstadt, Germany. Isopropyl alcohol (95%), 0.25N HCl, phenol red, sodium chloride, 0.1N NaOH, ethanol (95%), and benzoic acid were all obtained from UK Chemicals, Kumasi. Distilled water was obtained from the Department of Pharmaceutics Laboratory, KNUST.

### 2.2. Methods

#### 2.2.1. Collection and Extraction of Pectin from Unripe Peel of *Musa paradisiaca*

Unripe fruits of *Musa paradisiaca*, an intermediate French horn (oniaba), were obtained from the Ayeduase market in the Ashanti region and authenticated at the Department of Pharmacognosy, Faculty of Pharmacy and Pharmaceutical Sciences, Kwame Nkrumah University of Science and Technology (KNUST). The peels were removed, thoroughly washed, and cut into pieces. The peels were sun-dried for 7 days to a constant weight and comminuted with the aid of a blender into a powder form. The powders were divided into two and subjected to acid and alkaline extraction. The acidified water utilized was prepared by adding 5 mL of 1M HCl to 1000 mL of distilled water, while the alkaline water was prepared by adding 5 mL of 0.1 M NaOH to 1000 mL of distilled water. The ground powder (200 g) was transferred into a beaker, 250 mL of acidified water was poured into it, and the mixture was heated for 4 hours at 84°C. After the heating process, the heated sample was filtered, and the extracted pectin was precipitated by adding 250 mL of 95% ethanol to 100 mL of the extract. The mixture was stirred thoroughly and left to stand for 30 minutes. The gelatinous pectin flocculants were then skimmed off and filtered [17]. The sample was then dried in an oven at 30°C, and the resulting pectin was characterized. The exact process was followed for alkaline extraction.

#### 2.2.2. Determination of Percentage Yield of Unripe *Musa paradisiaca* Peel

The weight of the powdered peel of *Musa paradisiaca* (W1) and that of the dried, extracted pectin (W2) were determined using an analytical balance (Adam Equipment Ltd, model: ADP 2100, UK). The percentage yield was calculated as follows:

(1) $$\begin{array}{c} \text{percentage yield}=\frac{W2}{W1}\times 100\text{.} \end{array}$$

#### 2.2.3. Phytochemical Analysis on Extracted Pectin from Unripe *Musa paradisiaca* Peel

*(1) Test for Glycosides*. A mass of 0.2 g was warmed with 5 mL of dilute H_2_SO_4_ in a water bath for 2 minutes and filtered. The filtrate was made distinctively alkaline by adding four drops of 20% sodium hydroxide. A volume of 1 mL of each of Fehling's solutions A and B was added to the filtrate and warmed over a water bath for 2 minutes [18].

*(2) Test for Saponins*. A mass of 0.5 g of the pectin powder was shaken with 5 mL of distilled water in a test tube and filtered. The filtrate was shaken vigorously and allowed to stand for 5 minutes [18].

*(3) Test for Tannins*. 0.5 g of the pectin powder was boiled with 25 mL water for 5 minutes. It was then cooled and filtered. The extract was divided into two portions. A volume of 1 mL of the filtrate was pipetted, and 10 drops of 1% lead acetate solution were added. The precipitate formed was observed for its colour. A volume of 1 mL of the filtrate was pipetted, and 10 mL of distilled water was added. Afterwards, five drops of 1% ferric chloride solution were added [18].

*(4) Test for Phenols*. A 2 mL extract volume was treated with 4 drops of 0.1% ferric chloride solution [18].

*(5) Test for Pectin*. A volume of 9 mL of water was added to 1 g of pectin, stirred vigorously, heated, and cooled. A viscous liquid or a gel was formed. A volume of 1 mL of sodium hydroxide solution was added to 5 mL of 1% pectin solution. It was allowed to stand for 15 minutes, and a translucent, opaque gel was formed. A volume of 1 mL of hydrochloric acid was added to the gel precipitate formed. A colourless gel was formed, and white cotton-like precipitates were formed on boiling [19, 20].

#### 2.2.4. Characterization of *Musa paradisiaca* Pectin

*(1) Equivalent Weight of Musa paradisiaca Pectin*. A mass of 0.5 g of pectin was weighed into a 250 mL conical flask, and 5 mL of ethanol was added. A mass of 1 g of sodium chloride and 100 mL of distilled water were added. Six drops of phenol red were added. The resulting mixture was titrated against 0.1 N NaOH till an endpoint indicated by a purple colour was observed. The neutralized solution was stored to determine methoxyl content [21]. The result is expressed as

(2) $$\begin{array}{c} \text{equivalent weight}=\frac{\text{weight of pectin sample }\left(g\right)\times 1000mg}{\text{vol}.\text{ of alkali }\left(ml\right)\times \text{normality of alkali}}\text{.} \end{array}$$

*(2) Methoxyl Content of Musa paradisiaca Pectin*. Determination of methoxyl content was carried out by collecting the neutral solution from the determination of the equivalent weight. A volume of 25 mL of 0.25 N NaOH was added, and the solution was heated and stirred thoroughly and maintained at room temperature for 30 minutes. After 30 minutes, five drops of phenol red and a volume of 25 mL of 0.25 N HCl were added again, and it was titrated against 0.1 N NaOH until the colour changed from yellow to faint pink (endpoint) [21]. Methoxyl content is calculated as follows:

(3) $$\begin{array}{c} \text{methoxyl content }\left(\%\right)=\frac{\text{vol}.\text{ of alkali }\left(ml\right)\times \text{normality of alkali}\times 31\times 100}{\text{weight of pectin sample }\left(mg\right)}\text{,} \end{array}$$

where 31 indicates the group of methoxyl molecular weight.

*(3) Anhydrouronic Acid Analysis (AUA) of Musa paradisiaca Pectin*. Using the equivalent weight and methoxyl content value, AUA content would then be determined using the following equation:

(4) $$\begin{array}{c} \%\text{ AUA}=\frac{176\times 100}{Z}\text{,} \end{array}$$

where 176 is the molecular weight for AUA and *Z* is defined as the sample weight (mg) to NaOH milliequivalent (*µ*eq), which is the mL amount of NaOH from equivalent weight definition and methoxyl content defined as follows:

(5) $$\begin{array}{c} Z=\frac{\text{pectin weight }\left(mg\right)}{µeq\;\left(\text{equivalent weight}+\%\;MeO\right)}\text{.} \end{array}$$

*(4) Degree of Esterification (DE) of Musa paradisiaca Pectin*. The percentage degree of esterification of pectin was determined as follows:

(6) $$\begin{array}{c} \%\;DE=\frac{\%\text{MeO}\times 176\times 100}{\%\text{ AUA}\times 31}\text{.} \end{array}$$

*(5) Fourier-Transform Infrared (FTIR) Spectroscopy Analysis*. An FTIR spectrometer (PerkinElmer, UATR Spectrum 2, 941333, UK) was employed in the spectroscopic analysis of the acid- and alkaline-extracted *Musa paradisiaca* pectin.

#### 2.2.5. Formulation of Paracetamol Suspensions Using *Musa paradisiaca* Pectin as a Suspending Agent

Direct incorporation or dispersion method and levigation techniques were employed in preparing the paracetamol suspension, using *M. paradisiaca* pectin as a suspending agent and acacia gum as the standard at concentrations of 1% w/v and 2% w/v (Table 1). Each suspension was stored in an amber-coloured bottle, labelled accordingly, and stored at room temperature [22].

#### 2.2.6. Quality Control Tests on Suspensions

Each suspension was stored at room temperature; observations were made for 4 weeks to determine the flow rate/apparent viscosity, sedimentation rate, sedimentation volume, ease of redispersibility, and pH.

*(1) Sedimentation Volume (F)*. The suspension formulation (50 mL) was poured into 100 mL measuring cylinders separately, and the sedimentation volume was read after day one at weekly intervals for 3 weeks. Results were obtained for each formulation [20, 23, 24]. The suspension sedimentation volume was determined using the following equation:

(7) $$\begin{array}{c} F=\frac{Vu}{Vo}\times 100\%\text{,} \end{array}$$

where *Vu* is the ultimate volume and *Vo* is the initial volume of the suspension.

*(2) Flow Rate (f)*. The time taken for a 10 mL sample of suspension to flow through a 10 mL pipette was determined [20, 23, 24]. The flow rate was calculated using the following equation:

(8) $$\begin{array}{c} f=\frac{\text{volume of pipette }\left(ml\right)}{\text{time }\left(\sec\right)}\text{.} \end{array}$$

*(3) pH of Suspensions*. The suspensions were poured into 100 mL beakers and stirred just before immersing the electrodes of the pH meter, and the pH readings were taken using the pH meter. Triplicate results were obtained for each formulation [20, 23, 24].

*(4) Ease of Redispersibility*. The redispersibility of the suspension was evaluated qualitatively. The suspension was poured into 100 mL measuring cylinders separately and made to stand for 24 hours. After that, the cylinders were manually shaken, and the number of cycles required to redisperse the sediment was recorded [20, 23, 24].

*(5) Sedimentation Rate*. The suspensions were poured into 100 mL measuring cylinders separately and were allowed to stand undisturbed. The initial volume (50 mL) was recorded at time zero, and the volume of the sediment was recorded for each suspension at 10-minute intervals for one hour. The time and the volume recorded were used to determine the sedimentation rate graphically by plotting the volume of the sediment against the time [20, 23, 24].

#### 2.2.7. Drug Content Assay

An amount of 5 mL of the formulated suspensions was diluted with 45 mL of phosphate buffer, filtered, and topped up to 100 mL with phosphate buffer followed by a 1 in 50 dilution. The dilute solutions were spectrophotometrically assayed at 245 nm. The amount of paracetamol present was determined using a previously determined calibration curve, (*y*=806.29*x* − 0.0784, *R*^2^=0.9995). Triplicate determinations were obtained for each formulation.

#### 2.2.8. Statistical Analysis

GraphPad Prism (version 8) was used for the analysis. For all the data, the mean and standard deviation were calculated, respectively. Unpaired *t*-test results were used to calculate *P* values. *P* values of 0.05 or below were regarded as significant, while values higher than 0.05 were not.

## 3. Results and Discussion

### 3.1. Physicochemical Properties of *Musa paradisiaca*

The pectin yields obtained were 4.88% and 7.61% for the acid extracted pectin (ACEP) and alkaline extracted pectin (ALEP), respectively. The comparatively lower yields could be due to the geographical location of the plant, the time of harvesting, and the difference in extraction methods employed [25, 26]. Moreover, alkaline conditions cause instability in the backbone of the pectin molecule (galacturonic acid), and consequently, the pectin molecule tends to decompose, affecting alcohol precipitation of pectin [27]. Nevertheless, the yields correspond to the results obtained by Wandee et al. [15] that the alkaline extraction process produces higher yields at the same concentrations [15]. Acid extraction has been reported to extract various types of pectins, particularly protopectins, while alkaline extraction has been reported to extract a high amount of pectins compared to neutral conditions [27, 28].

Phytochemical screening revealed the presence of glycosides, tannins, saponins, and phenols in both ACEP and ALEP (Table 2). These secondary metabolites have been reported to be responsible for the antioxidant and antimicrobial properties of natural polysaccharides such as pectin [29]. The extracted pectins also complied with pectin identification requirements [30] (Table 3).

#### 3.1.1. Characterization of *Musa paradisiaca* Pectin

Characterizing pectin is essential in ascertaining purity and identifying better pectin sources. Equivalent weight measures the free galacturonic acid content in pectin. The amount of free galacturonic acid present in pectins impacts its gelling properties and depends on the type of plant, quality of raw materials, and method of extraction [27]. The equivalent weights of ACEP and ALEP were 1666.667 ± 0.032 mg/mol and 3125.000 ± 0.065 mg/mol, respectively (Table 4). The high equivalent weight of ALEP may translate into higher gelling properties as high equivalent weight polymers have a higher gel-forming effect [31]. Furthermore, the low equivalent weight of ACEP may indicate pectin degradation in the HCl medium, as well as reduced quantities of free acids, as high acidity may cause polymerization of pectin, resulting in reduced free acids [31, 32].

The gelling abilities of pectins, in addition to its set times, are determined by the methoxyl content [33]. Values ≥7% are classified as a high methoxyl content, and it is affected by the source of pectin and the extraction technique employed [34]. ACEP and ALEP could be described as containing high methoxyl contents, as 15.69% and 8.585% were recorded, respectively. This implies that both pectins could form high-sugar gels and disperse readily in aqueous media [35]. This indicates their potential suitability as gelling agents in food and beverage industries and as possible binders in the formulation of immediate-release pharmaceutical tablets.

Anhydrouronic acid (AUA) content is a determinant of pectin quality, and its levels impact the structure and texture of the pectin gel formed [28]. It has been reported that an AUA content of <65% is an indication of high amounts of protein, starch, and sugar [32, 36, 37]. The AUA content obtained from ACEP and ALEP was 99.62% and 54.154%, respectively (Table 3). This indicates that ACEP is sufficiently pure compared to ALEP which may have a high amount of impurities.

The degree of esterification (DE) of ACEP and ALEP was found to be 89.40% and 90.01%, respectively, which translated into a high degree of esterification for the extracted pectins and were comparable to other studies [35] (Table 3). The high degree of esterification also establishes that pectin has high gelation and emulsification properties [38]. This further corroborates its potential uses in food and beverage industries and as potential emulsifying agents in the formulation of pharmaceutical emulsions.

The moisture content is vital in the pharmaceutical industry because it influences powders' flow properties and microbial growth [39, 40]. The moisture content was 11.785% for ACEP and 6.614% for ALEP (Table 4). As pharmaceutical excipients, the moisture contents were within pharmacopoeia standards as the British Pharmacopoeia prescribes that moisture content should be below 15% w/w [41, 42]. Nevertheless, the results suggest that ACEP contains higher moisture content, thus a high tendency of microbial growth and poor flow properties compared to ALEP when stored for longer periods.

The ash content represents the purity of pectin. The lower the ash content, the higher the purity of pectin [36]. The ash contents obtained, 2.56% for ACEP and 4.06% for ALEP, were comparatively lower than those in other reported literature studies, indicating a higher quality of the extracted pectins.

The amount of protein from ACEP and ALEP was 8.123% w/v and 11.633% w/v, respectively. It has been demonstrated that proteinaceous elements linked to pectin polymer chains play direct roles in the activating and stabilizing properties of the pectin powder, particularly in pharmaceutical emulsions [43, 44]. The relatively higher amount of protein in ALEP may be due to increased covalent linkages between proteins and pectin, resulting in coprecipitation by ethanol [45, 46]. This further corroborates the AUA content findings that ACEP was relatively purer, where protein hydrolysis could account for lower crude protein contents [45].

**Figure 1:**
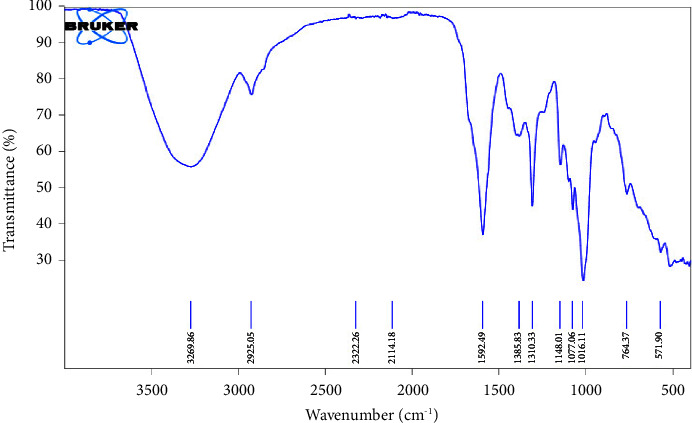
FTIR spectrum of ACEP.

**Figure 2:**
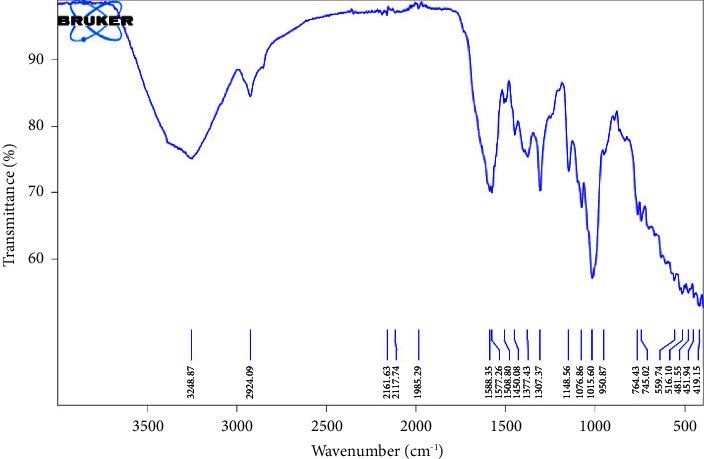
FTIR spectrum of ALEP.

The FTIR spectra (Figures 1 and 2) identified and characterized the components in ACEP and ALEP. The principal bands for ACEP were shown at 3269.86 cm^−1^, 2925.05 cm^−1^, 2114.18 cm^−1^, 1592.49 cm^−1^, 1310.33 cm^−1^, and 1148 cm^−1^, which were assigned to the O-H stretch, C-H stretching vibrations, C≡C terminal alkyne, carboxylate (-COO) + moiety, C-N amine stretch, and the C-O stretch of tertiary alcohol, respectively. In similitude, ALEP showed the corresponding principal bands at 3248.87 cm^−1^, 2924.09 cm^−1^, 2117.74 cm^−1^, 1577.25 cm^−1^, 1307.37, and 1148.56 cm^−1^. The FTIR spectra obtained corresponded with other published literature studies [47, 48]. Furthermore, ALEP contained more ester groups than ACEP, which could be accounted for the hydrolysis of the ester groups by HCl employed in its extraction [45].

### 3.2. Quality Control of Formulated Suspensions

#### 3.2.1. pH of Suspensions

pH analysis is essential in the stability evaluation of suspension dosage forms [22]. Reasonably constant weakly acidic pH with no significant changes (*P* > 0.05) was observed for all the suspensions during 4 weeks (Table 5). Thus, it can be inferred that there will be no pH instability issues when the formulations are stored for prolonged periods. Moreover, weakly acidic pH can prevent microbial growth, preventing degradation and enhancing stability during the shelf life [22]. This was further confirmed by the absence of any unwanted physical changes, including crystal growth formation and caking after critical observation.

#### 3.2.2. Ease of Redispersibility of Suspensions

**Figure 3:**
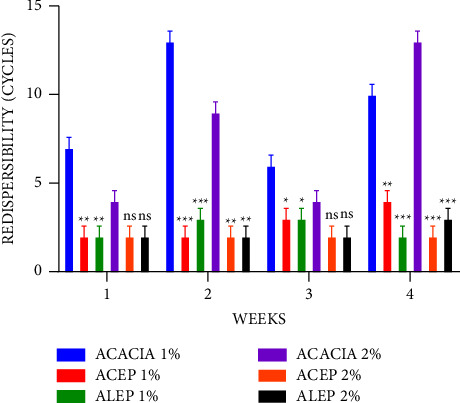
Redispersibility of acacia and pectin suspensions using Student's two-tailed test. ^*∗*^*P* ≤ 0.05, ^*∗∗*^*P* ≤ 0.01, ^*∗∗∗*^*P* ≤ 0.001, and *P* > 0.05 not significant (ns).

**Figure 4:**
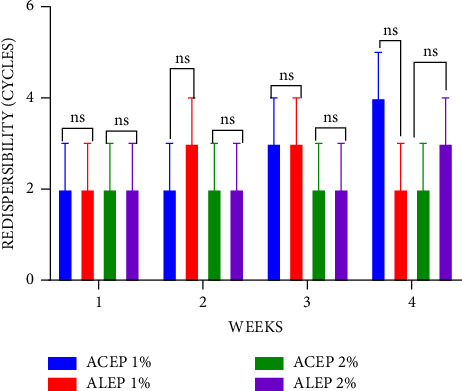
Redispersibility of ALEP and ACEP suspensions using Student's two-tailed test. *P* > 0.05 is not significant (ns).

The ease of redispersibility is a function of the number of cycles required to convert the sediment to a homogeneous suspension when rotated through 180°. The fewer the cycles, the better the ease of redispersion and the better the suspension [49–51]. During 4 weeks, 1% ACEP and 1% ALEP demonstrated significant (*P* < 0.05) differences in the ease of redispersibility compared with 1% acacia. However, when the concentrations were increased to 2%, there was a nonsignificant (*P* ≥ 0.05) difference in the ease of redispersibility during the first and third weeks when the suspensions were compared (Figure 3). When the ALEP and ACEP suspensions alone are compared in Figure 4, there was a nonsignificant (*P* > 0.05) difference in their ease of redispersibility at all concentrations, which can be inferred that the method of extraction does not affect the conversion of sediments to a homogeneous suspension.

#### 3.2.3. Flow Rate of Suspensions

**Figure 5:**
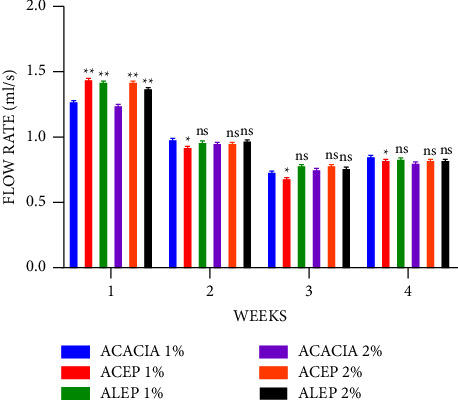
Flow rate of acacia and pectin suspensions using Student's two-tailed test. ^*∗∗*^*P* ≤ 0.01, ^*∗*^*P* ≤ 0.05, and *P* > 0.05 not significant (ns).

During the 4-week observation period, the suspensions exhibited pseudoplastic non-Newtonian flow, which is an ideal property of suspensions allowing for accurate doses to be measured (Figure 5) [50, 51]. The flow rates of all the formulated suspensions were observed to decrease with time, a natural characteristic of gums and pectins accounted for by autocatalytic hydrolysis [52]. The lack of physical changes during the study period suggests that this process had no detrimental effect on suspension stability. Moreover, as the concentrations were increased from 1% to 2%, the flow rate decreased, which is an ideal property of suspending agents to ensure easy pourability [53, 54].

**Figure 6:**
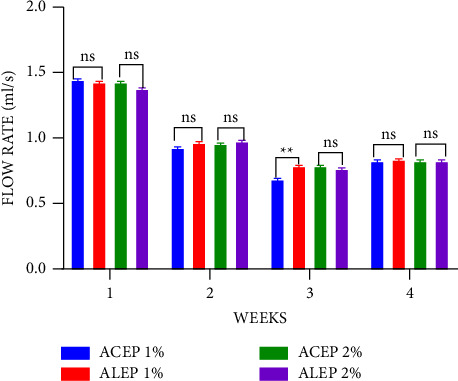
Flow rate of ALEP and ACEP suspensions using Student's two-tailed test. *P* > 0.05 is not significant (ns).

The flow rates of the extracted pectins were significantly higher (*P* ≤ 0.01) than those of acacia during the first week at all concentrations; however, in the subsequent weeks, only 1% ACEP had a significantly higher flow rate than 1% acacia, with the rest recording a nonsignificant difference. This suggests that the extracted pectins were comparable to the acacia gum. However, except for the third week of storage, when the ACEP and ALEP suspensions were compared, there was no significant difference between them, further suggesting that the method of extraction does not affect the flow rate of unripe *Musa paradisiaca* peels pectin (Figure 6).

#### 3.2.4. Sedimentation Volume and Sedimentation Rate of Suspensions

**Figure 7:**
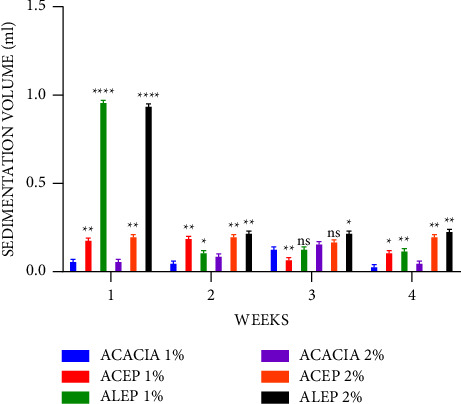
Sedimentation volume of acacia and pectin suspensions using Student's two-tailed test. ^*∗∗∗∗*^*P* ≤ 0.0001, ^*∗∗*^*P* ≤ 0.01, ^*∗*^*P* ≤ 0.05, and *P* > 0.05 not significant (ns).

The sedimentation volume is essential for suspensions' quality assurance and physical stability. Values typically range from less than 1 to 1, with sedimentation values nearing 1 being attributed to better suspending properties [50, 55, 56, 57]. During the first week of this study, it was observed that the sedimentation volume of ALEP was significantly higher (*P* ≤ 0.0001) than that of acacia at both 1% and 2% concentrations and approached 1 (Figure 7). ACEP was also significantly higher than acacia at both concentrations (*P* ≤ 0.01). Similar observations were made across 4 weeks, with a significant difference being observed between the extracted pectins and the acacia gum suspensions. It can therefore be inferred that pectin from unripe *Musa paradisiaca* peels is a suitable suspending agent.

**Figure 8:**
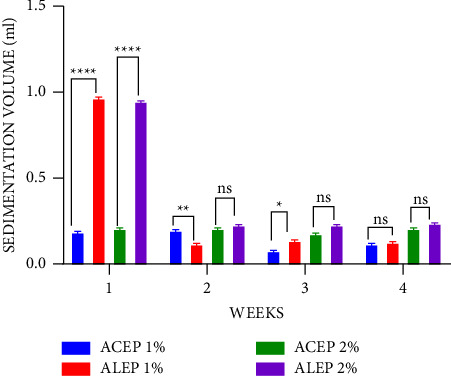
Sedimentation volume of ALEP and ACEP suspensions using Student's two-tailed test. ^*∗∗∗∗*^*P* ≤ 0.0001, ^*∗∗*^*P* ≤ 0.01, ^*∗*^*P* ≤ 0.05, and *P* > 0.05 not significant (ns).

A comparison of the sedimentation volume of ALEP and ACEP revealed that, during the first week, ALEP was significantly higher (*P* ≤ 0.0001) than ACEP at all concentrations; however, a nonsignificant (*P* > 0.05) difference was observed in the fourth week (Figure 8). This may suggest that the alkaline extracted method produces pectins with better suspending properties.

**Figure 9:**
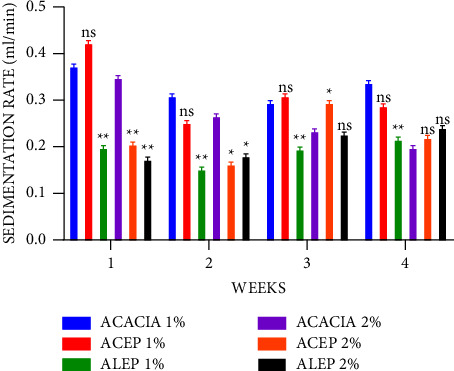
Sedimentation rate of acacia and pectin suspensions using Student's two-tailed test. ^*∗∗*^*P* ≤ 0.01, ^*∗*^*P* ≤ 0.05, and *P* > 0.05 not significant (ns).

**Figure 10:**
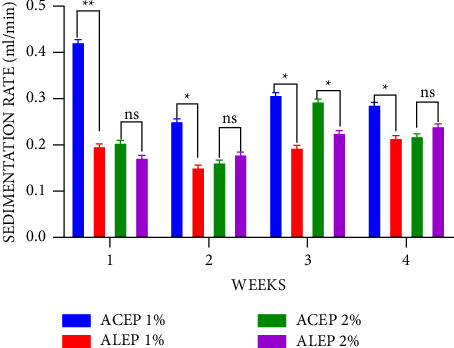
Sedimentation rate of ALEP and ACEP suspensions using Student's two-tailed test. ^*∗∗*^*P* ≤ 0.01, ^*∗*^*P* ≤ 0.05, and *P* > 0.05 not significant (ns).

The sedimentation rate is inversely proportional to the quality of the suspension; therefore, the slower the rate of sedimentation, the better the suspending property. From the results in Figure 9, it was observed that there was a general decrease in the sedimentation rate as the concentration increased from 1% to 2% for all the suspensions corroborating the findings that concentration affects the sedimentation rate [22]. It was also observed that at 1%, ALEP exhibited a significantly slower sedimentation (*P* ≤ 0.01) rate than 1% acacia throughout the 4 weeks. 2% ACEP also exhibited a significantly lower sedimentation rate from weeks 1 to 3, while 2% ALEP only exhibited similar qualities in weeks 1 and 2. This suggests that the extracted pectin, mainly ALEP, had better suspending properties than acacia gum. At a 1% concentration, ALEP exhibited a better suspending activity when the sedimentation rate of ACEP and ALEP was compared (Figure 10). This supports earlier claim that the alkaline extraction method may yield pectins with better suspending properties.

#### 3.2.5. Drug Assay

According to the British Pharmacopoeia, the computed amount of drug substance in each of the dosage units should fall between the ranges of 85.0% and 115.0%, with none deviating outside the 75.0%– 125.0% range [42]. The suspension passed the drug content test since every dose that was analysed contained the necessary amount of the active component (Table 6). This suggests that each volume will supply the required quantity of the active component and prevent unwanted overdosing and underdosing for the patient. The formulation procedures were also appraised to be effective and efficient.

## 4. Conclusion

Pectin extracted from plantain peels had suspending properties at 1% w/v and 2% w/v concentrations, comparable to Acacia gum. Moreover, the alkaline extraction technique may produce pectin with a better suspending ability than the acid extraction technique.

## Figures and Tables

**Table 1:** Master formula for the preparation of paracetamol suspension.

Ingredients	Quantities
Paracetamol powder	5 g
Suspending agents (1% w/v or 2% w/v)	1 g or 2 g
Benzoic acid (0.1% w/v)	0.1 g
Purified water	100 mL

The suspending agent was *M. paradisiaca* pectin or acacia gum.

**Table 2:** Phytochemical profile of acid- and alkaline-extracted *Musa paradisiaca* pectin.

Phytochemicals	Observation	Inference
Glycosides	Presence of brick-red precipitate	Glycoside is present
Saponins	A froth that persisted for 5 minutes	Saponin is present
Tannins	A light brown precipitate formed, which persisted after the addition of 5 drops of 1% ferric chloride solution	Tannin is present
Phenols	Bluish-black colour	Phenol is present

**Table 3:** Identification test for pectin in acid and alkaline extracts from *Musa paradisiaca* peels.

Samples	Observation	Result
ACEP	A translucent, opaque gel formed, and upon the addition of 1 mL of HCl, a colourless gel was observed and a white cotton-like precipitate formed on boiling	Pectin is present
ALEP	A translucent, opaque gel formed, and upon the addition of 1 mL of HCl, a colourless gel was observed and a white cotton-like precipitate formed on boiling	Pectin is present

**Table 4:** Characterization of pectin from unripe *Musa paradisiaca* peels.

Parameters	ACEP	ALEP
Equivalent weight (mg/mol)	1666.667 ± 0.032	3125.000 ± 0.065
Methoxyl content (%)	15.690 ± 0.034	8.585 ± 0.006
Anhydrouronic acid content, AUA (%)	99.620 ± 0.047	54.154 ± 0.032
Degree of esterification, DE (%)	89.400 ± 0.006	90.010 ± 0.072
Moisture content (%)	11.785 ± 0.015	6.614 ± 0.006
Ash content (%)	2.560 ± 0.007	4.060 ± 0.006
Crude protein (%)	8.123 ± 0.009	11.633 ± 0.003

Each value is expressed as the mean ± SD (*n* = 3).

**Table 5:** pH of acacia and pectin suspensions.

Weeks	(Acacia) 1%	ACEP 1%	ALEP 1%	Acacia 2%	ACEP 2%	ALEP 2%
Week 1	3.58 ± 0.012	3.51 ± 0.033^ns^	3.45 ± 0.032^ns^	3.70 ± 0.029	3.67 ± 0.009^ns^	3.43 ± 0.006^ns^
Week 2	2.67 ± 0.009	2.93 ± 0.045^ns^	3.04 ± 0.012^ns^	2.91 ± 0.003	3.02 ± 0.038^ns^	3.81 ± 0.038^ns^
Week 3	3.06 ± 0.015	3.17 ± 0.006^ns^	3.96 ± 0.006^ns^	3.22 ± 0.006	3.18 ± 0.006^ns^	3.94 ± 0.006^ns^
Week 4	3.14 ± 0.006	3.38 ± 0.037^ns^	3.18 ± 0.010^ns^	3.39 ± 0.009	3.43 ± 0.021^ns^	4.19 ± 0.035^ns^

Each value is expressed as the mean ± SD (*n* = 3). *P* > 0.05 nonsignificant (^ns^).

**Table 6:** Assay of acacia and pectin suspensions.

Formulations	% drug content
Acacia 1%	103.78 ± 0.030
ACEP 1%	103.29 ± 0.030
ALEP 1%	99.32 ± 0.080
Acacia 2%	101.55 ± 0.110
ACEP 2%	99.82 ± 0.100
ALEP 2%	102.3 ± 0.020

## Data Availability

The data used to support the findings of this study have been included in the article and can be made available from the corresponding author on request.
